# The *Schistosoma mansoni* Tegumental-Allergen-Like (TAL) Protein Family: Influence of Developmental Expression on Human IgE Responses

**DOI:** 10.1371/journal.pntd.0001593

**Published:** 2012-04-03

**Authors:** Colin M. Fitzsimmons, Frances M. Jones, Alex Stearn, Iain W. Chalmers, Karl F. Hoffmann, Jakub Wawrzyniak, Shona Wilson, Narcis B. Kabatereine, David W. Dunne

**Affiliations:** 1 Department of Pathology, University of Cambridge, Cambridge, United Kingdom; 2 Institute of Biological, Environmental and Rural Sciences, Aberystwyth University, Aberystwyth, United Kingdom; 3 Vector Control Division, Ugandan Ministry of Health, Kampala, Uganda; Federal University of Minas Gerais, Brazil

## Abstract

**Background:**

A human IgE response to Sm22.6 (a dominant IgE target in *Schistosoma mansoni*) is associated with the development of partial immunity. Located inside the tegument, the molecule belongs to a family of proteins from parasitic platyhelminths, the Tegument-Allergen-Like proteins (TALs). In addition to containing dynein-light-chain domains, these TALs also contain EF-hand domains similar to those found in numerous EF-hand allergens.

**Methodology/Principal Findings:**

*S. mansoni* genome searches revealed 13 members (SmTAL1-13) within the species. Recent microarray data demonstrated they have a wide range of life-cycle transcriptional profiles. We expressed SmTAL1 (Sm22.6), SmTAL2, 3, 4, 5 and 13 as recombinant proteins and measured IgE and IgG4 in 200 infected males (7–60 years) from a schistosomiasis endemic region in Uganda. For SmTAL1 and 3 (transcribed in schistosomula through adult-worms and adult-worms, respectively) and SmTAL5 (transcribed in cercariae through adult-worms), detectable IgE responses were rare in 7–9 year olds, but increased with age. At all ages, IgE to SmTAL2 (expressed constitutively), was rare while anti-SmTAL2 IgG4 was common. Levels of IgE and IgG4 to SmTAL4 and 13 (transcribed predominantly in the cercariae/skin stage) were all low.

**Conclusions:**

We have not measured SmTAL protein abundance or exposure in live parasites, but the antibody data suggests to us that, in endemic areas, there is priming and boosting of IgE to adult-worm SmTALs by occasional death of long-lived worms, desensitization to egg SmTALs through continuous exposure to dying eggs and low immunogenicity of larval SmTALs due to immunosuppression in the skin by the parasite. Of these, it is the gradual increase in IgE to the worm antigens that parallels age-dependent immunity seen in endemic areas.

## Author Summary

Examining the genome of the parasitic worm *Schistosoma mansoni*, we have identified and defined the structure of a family of 13 allergen-like proteins from the outer layer (tegument) of the organism. We term these molecules the *S. mansoni* Tegument-Allergen-Like proteins (SmTALs). During *S. mansoni* infection the human host is exposed to skin-invading larvae, adult-worms (living in the blood) and to parasite eggs. These life-stages have very different sizes, tissue composition and gene expression. We have produced 6 SmTAL proteins with different life-cycle transcriptional patterns and measured IgE antibody responses to them in 200 infected males from an *S. mansoni* endemic area. The binding of IgE to foreign proteins is important in allergy but also in defence against parasitic worms. Our results suggest that, in these endemic areas, there is priming and boosting of IgE responses to adult-worm SmTALs by the occasional death of long-lived worms, desensitization to egg SmTALs due to continuous exposure to dying eggs and low immunogenicity of larval SmTALs perhaps due to immunosuppression in the skin by the parasite. Schistosome infection is a major health problem in many countries. Our work provides insight into what provokes and controls the antibody responses associated with human immunity to this parasite.

## Introduction

Schistosomiasis is caused by infection with parastic worms of the genus *Schistosoma*, with *S. mansoni*, *S. haematobium* and *S. japonicum* being the predominant species to affect humans. It is a major public heath problem in many developing countries and, amongst parasitic diseases, is second only to malaria in its impact on human health [1]. In areas highly endemic for schistosomiasis, people can remain infected for most of their lives, but as they get older, their worm burden is reduced and they become more resistant to re-infection [2]. In these communities an age-dependent immunity develops, targeted presumably at vulnerable stages of the parasite life-cycle.

Infection occurs in fresh water when microscopic cercariae penetrate exposed skin. This initiates conversion into parasitic schistosomula that migrate via the lungs to the liver, mature, pair and then live for many years in small veins in the abdomen. It has been estimated that in *S. mansoni* endemic areas, adult-worms live in the human blood stream for 7–9 years [3]. Each female worm lays hundreds of eggs per day that are either excreted from, or become trapped in host tissue to die over a period of weeks [4]. Excreted eggs hatch in water to release miracidia that penetrate fresh water and as sporocysts, undergo two distinct phases of asexual reproduction, before emerging again as infectious cercariae.

A number of epidemiological studies have correlated the human IgE response against schistosomula or adult-worms with immunity [5]–[8]. By monitoring re-infection after therapeutic drug treatment, it has been shown that people with high levels of parasite-specific IgE are significantly less likely to become re-infected with S. *haematobium*, *S. japonicum* or *S. mansoni* [5]–[9]. IgG4 on the other hand, is an antibody that blocks the effects of IgE [10] and a number of epidemiological studies associate elevated parasite-specific IgG4 with susceptibility to re-infection [5], [6], [9]. The mechanisms linking high levels of anti-parasite IgE to human immunity has yet to be demonstrated *in vivo*. However, it has been shown that a number of different immune effector cells can kill schistosomula in an IgE dependent manner *in vitro* [11].

Dunne and colleagues report that the dominant target for human IgE in *S. mansoni* is a protein, Sm22.6 [8]. They showed that an IgE response to this molecule was correlated with resistance to re-infection in treatment studies in Kenya and Uganda [8], [12]–[14]. The orthologs, Sj22.6 in *S. japonicum* and Sh22.6 in *S. haematobium*, are also human IgE antigens [15], [16]. They belong to a family that includes other well-characterized *S. mansoni* proteins Sm21.7, Sm20.8 and Sm21.6 [17]–[20] and homologous proteins across parasitic platyhelminth species [17]. Collectively, these proteins are all characterized by a C-terminal region resembling a dynein light chain (DLC)-1 domain [17] and two N-terminal EF-hand motifs (Pfam accession No. PF00036). The inclusion of EF-hand motifs gives them close structural similarity with one of the most common groups of clinical allergens, the EF-hand allergens [15], [21]. We, therefore, term this parasite protein family the Tegument-Allergen-Like (TAL) proteins [14], [22], [23]. Those TALs that have been localized [12], [24]–[28] have all been strongly associated with the tegument, a syncytial structure that forms the outer layer of the organism.

Although the SmTAL proteins are all similar in domain structure and size, they have different expression profiles throughout the complex schistosome life-cycle [24], [25], [29] and we have suggested that these differences may influence the dynamics of specific antibody responses in the human host [29]. In a previous report [29], we noted that Sm22.6 (SmTAL1), Sm21.7 (SmTAL2) and Sm20.8 (SmTAL3) were abundant in adult-worms, but only SmTAL2 was present in parasite eggs [29]. We showed that levels of IgE to worm antigens SmTAL1 and SmTAL3 were high in a population from an *S. mansoni* endemic area [29], and boosted when worms were disrupted *in vivo* by chemotherapy. In contrast, levels of circulating IgE to the egg antigen SmTAL2 were low during infection and did not increase with treatment [29].

The *S. mansoni* genome is fully sequenced and annotated [30] and we have searched the public databases exhaustively for other putative SmTAL family members (as defined above). Now, having confirmed the transcription and sequence of candidate molecules we conclude that there are 13 TALs in the *S. mansoni* genome, SmTAL1–13. Fitzpatrick *et al.* [31] recently constructed a comprehensive *S. mansoni* long-oligonucleotide microarray and, with their data obtained using RNA from 15 different life-cycle stages, it is possible to examine developmental expression of virtually any transcript. Using this resource, we report that some SmTALs are transcribed constitutively throughout the *S. mansoni* life-cycle, some are restricted to expression only within the definitive host, two are predominantly expressed in cercarial/skin parasitic stages and one is primarily expressed only in the miracidia/snail stages. We have chosen 6 SmTALs displaying diverse transcription patterns, expressed recombinant proteins and used these antigens to examine SmTAL-specific IgE and IgG4 responses in a cross-sectional cohort of 200 *S. mansoni* infected males from a highly endemic region of Uganda.

## Materials and Methods

### Finding SmTALs in the *S. mansoni* genome

The nucleotide and amino acid sequences of the well characterized SmTAL1 (XP_002575844, Smp_045200.1), SmTAL2 (XP_002569898, Smp_086480.1) and SmTAL3 (XP_002569900, Smp_086530.1) were used in BLAST searches of NCBI/GenBank, UniProt and GeneDB databases to identify other potential SmTALs. The criteria for SmTAL identification were overall sequence similarity (E<e-16), the presence of a predicted N-terminal EF_HAND_2 domain (EMBL/EBI IPR018249) in combination with a C-terminal dynein light chain domain (IPR001372) or sequences with N and C-terminal domains which were highly similar to those in SmTAL1, 2 or 3 but which were not annotated as EF-hand or DLC domains in the public databases. As a result of these searches, ten further SmTALs were identified: SmTAL4 (Smp_169190.1), SmTAL5 (Smp_195090.1), SmTAL6 (Smp_072620.1), SmTAL7 (Smp_042140.1), SmTAL8 (Smp_086470.1), SmTAL9 (Smp_077310.1) SmTAL10 (Smp_074460.1), SmTAL11 (Smp_169200.1), SmTAL12 (Smp_045010.1) and SmTAL13 (Smp_042150.1), of which SmTAL8 has recently been expressed and characterized by Lopes *et al.* as Sm21.6 [20]. All 13 SmTAL sequences were then used to repeat the BLAST searches. No further family members were identified.

### Parasite mRNA

Shedding of cercariae was stimulated by exposure of snails to a bright light. Parasites were then mechanically transformed into schistosomula as previously described [31], [32]. Total RNA was isolated as previously described [32].

### Microarray transcription profiles

Data from the 37,632 element *S. mansoni* long-oligonucleotide microarray studies of Fitzpatrick et al were interrogated to find the transcription pattern of the 13 SmTALs in 13 life-cycle stages [31]. A full set of raw and normalized data is available via Array express [33] under the experimental accession number E-MEXP-2094.

### Cloning and sequencing SmTAL transcripts

Using *S. mansoni* genome data [34] to design primers (Table S1), the full coding sequences of SmTAL4, 5, 8 and 13 were amplified by PCR using cDNA prepared from total RNA obtained from cercariae (SmTAL4 and 13), eggs (SmTAL6) or 7 wk mixed sex adult-worms (SmTAL5, 7, 8, 9, 10, 11 and 12). The resultant products were directly cloned into the vector pCR4-TOPO TA (Invitrogen) and transformed into TOP10 *E. coli* cells (Invitrogen). Recombinant plasmids were then isolated (MiniPrep, Qiagen) and sequenced (DNA Sequencing Facility Cambridge University Dept. Genetics). The mRNA coding sequence of the SmTAL4–7 and 9–13 were uploaded to EMBL (Acc Nos HE616805–HE616812).

### Expression and purification of recombinant SmTAL proteins

Recombinant SmTAL1, 2 and 3 proteins were prepared as described previously [29]. The full coding sequences of SmTAL4, 5, and 13 were amplified using the plasmids (above) as template and specific primers with appended restriction sites (Table S2). Amplicons were ligated into the pGEX-4T-3 expression vector (GE healthcare) between the BamHI and EcoRI (SmTAL4 and 5) or BamHI and XhoI (SmTAL13) restriction sites and constructs were sequenced to verify inserted sequences. GST-SmTAL fusion proteins (5′ GST) were expressed in *E. coli*, isolated by affinity chromatography on Glutathione-agarose and cleaved with thrombin as previously described [29]. GST was removed by passing each digest of fusion protein through Q-Sepharose anion exchange beads (Amersham Bioscience) equilibrated with 50 mM Tris/HCl pH 8.0 containing 10 mM reduced glutathione (SmTAL4 and 5) or by cleaving the fusion protein on glutathione agarose (SmTAL13). Thrombin was removed by addition of benzamadine-agarose beads (Sigma). In each case the absence of contaminating GST was confirmed by ELISA using rabbit-anti-GST antisera (Sigma) and HRP-conjugated anti-rabbit IgG (Sigma).

### Study population

The study cohort were inhabitants of Musoli, a fishing community on Lake Victoria. 228 males infected with *S. mansoni* (7 to 76 years, mean age 25) were randomly selected after initial parasitological screening of the entire Musoli population. Quantitative parasitology on each individual was carried out on 5 stool samples collected on different days (2 Kato-Katz thick smear slides per sample). The median pre-treatment egg count for the selected cohort was 446 eggs per gram of faeces (epg) (range 3 to 7083 epg). In this report we focus on 200 members of the cohort who were under 60 y and who donated blood before and 9 wks after they received 40 mg/kg praziquantel (PZQ).

### Ethics statement

Ethical clearance was obtained from the Uganda National Council of Science and Technology (ethics committee for Vector Control Division, Ugandan Ministry of Health). Consent forms were translated into the local language and informed written consent was obtained from all adults and from the parents/legal guardians of all children under 15.

### ELISA

IgE and IgG4 levels were measures by isotype specific ELISA essentially as described previously [35]. Saturating coating concentration for each recombinant was determined in advance using either in-house rabbit anti-sera, or a coating inhibition assay [36]. 384-well high-binding microplates (Greiner bio-one Ltd.) were washed with water and coated with 15 µl SmTAL in 0.1 M sodium bicarbonate pH 9.6 overnight at 4°C. Coating concentrations were 5.4, 2.7, 5.5, 6.2, 4.8 and 6.0 µg/ml for SmTAL1, 2, 3, 4, 5 and 13 respectively. The wells were washed 4 times with PBS containing 0.03%(v/v) Tween 20 using a BioTek ELx405 plate washer and blocked by incubating for 1 h with 1% (w/v) milk powder (Marvel) in PBS. To measure IgE, plasma was diluted 1∶20 with 10%(v/v) fetal calf serum (FCS) in ELISA buffer (PBS containing 0.1%(w/v) milk powder and 0.05% (v/v) Tween 20). To measure IgG4 plasma was diluted 1∶200 with 1%(v/v) FCS in ELISA buffer. Following overnight incubation at 4°C, wells were washed again and incubated for 4 h with ELISA buffer containing 0.5 µg/ml biotinylated mouse anti-human IgE (Clone G7-18, Pharmingen) or 0.5 µg/ml biotinylated mouse anti-human IgG4 (Clone JDC-14, Pharmingen). After washing, wells were incubated for 1 h with streptavidin/biotinylated–horse radish peroxide complex (Mast Group Ltd.), diluted 1∶3000 in ELISA buffer and then washed again. The assay was developed with 68 µl o-phenylenediamine substrate solution (Sigma) and stopped with 17 µl of 2 M sulphuric acid as required. A standard curve was generated by coating a serial dilution of human myeloma proteins, IgE (Calbiochem) or IgG4 (Sigma) as appropriate, and plasma samples from 26 uninfected European donors were included in each assay. The plates were read using a Powerwave HT reader (BioTek Instruments Inc.) at a test wavelength of 490 nm and a reference wavelength of 630 nm. The OD values from the myeloma data were used by the ELISA reader software to generate standard curves using 5-parameter logistic regression.

### Reverse transcription (RT)-PCR analysis of cercarial heads and tails

After mechanical transformation, cercarial heads and tails were separated by centrifugation on Percoll (Sigma) as described previously [37]. The levels of mRNA for Sm ß-actin and SmTAL4, 5 and 13 were determined by RT-PCR as described [38] using primer sequences included in Table S3.

### Immunostaining of cercarial sections with SmTAL4-specific antiserum

SmTAL4-specific rat antiserum was a gift from Dr Jamal Khalife (Institut Pasteur de Lille). Sections (8 µm) were cut from frozen blocks of cercariae mounted in OCT (Sakura Ltd) and placed on 1 mm thick SuperFrost slides (VWR Int.) After thawing, they were fixed for 5 min in ice-cold acetone and washed with PBS (used for all subsequent washes), blocked with goat sera for 30 min and washed again. Sections were then incubated with SmTAL4-specific rat antiserum (1∶200 in PBS) for 1 hr, washed, incubated for 1 hr with anti-rat TRITC (1∶100, eBioscience), washed again and finally counterstained with DAPI (200 ng/ml) for 30 mins. The sections were mounted with Fluoromount (Sigma) and prepared for epifluorescent imaging. A negative control slide was prepared by pre-absorbing the antiserum with recombinant SmTAL4 antigen (100 µg/ml) before use. Stained slides were viewed immediately using a Zeiss Axiophot fluorescent microscope.

## Results

### 13 SmTALs are transcribed from the *S. mansoni* genome

**Figure 1:**
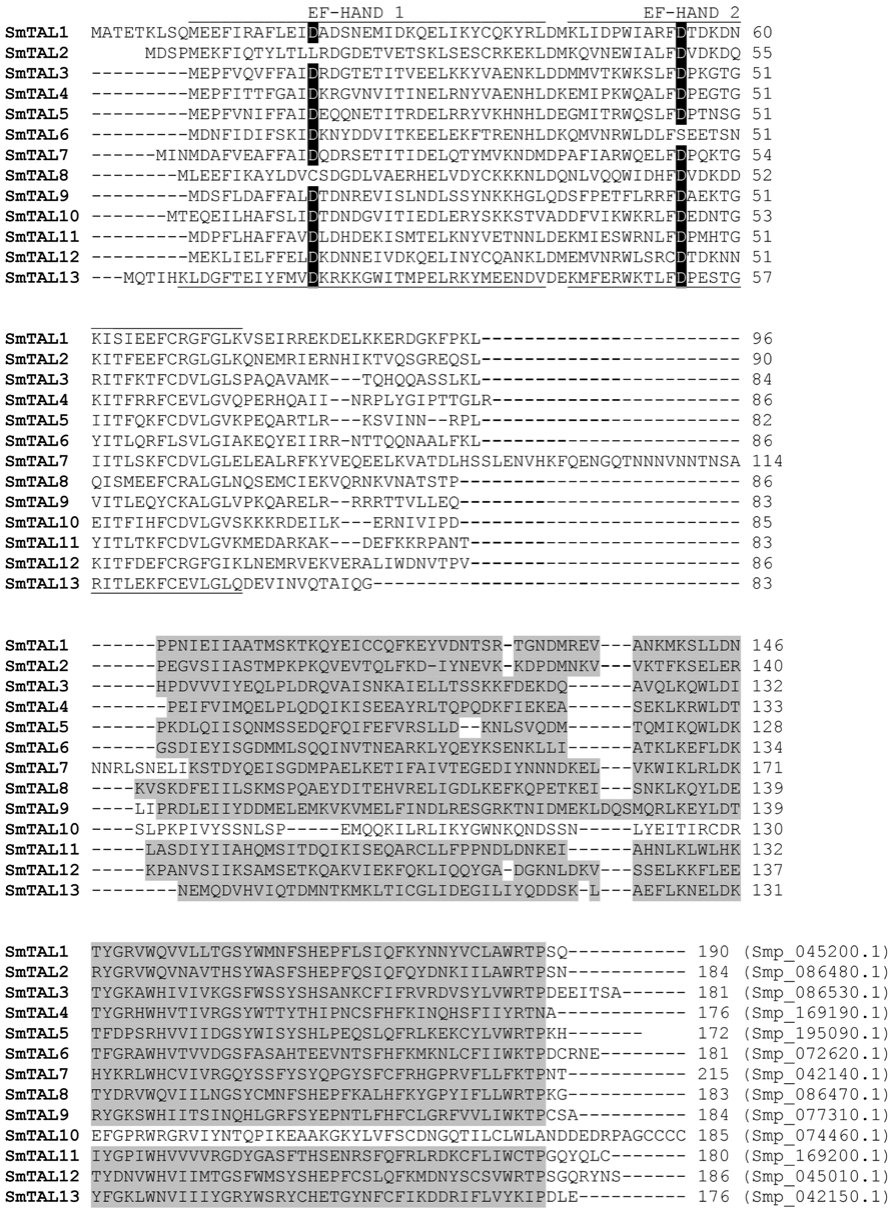
Alignment of amino acids sequences of the SmTAL family. Helix-loop-helix EF-hand domains (Pfam00036) are indicated. The canonical aspartic acid residues at the start of the loop are denoted D on black background. Residues in the dynein light chain domain (EMBL/EBI IPR001372) are shaded grey. Alignment was performed using Clustal W.

Using published sequences of SmTAL1 2, and 3 for extensive searches of the public databases, and visual examination of aligned candidate sequences, we identified 10 more members of the SmTAL family (SmTAL4–13) including the recently characterized SmTAL8 [20]. Using parasite material, we confirmed that all 10 are transcribed and their sequences are essentially identical to the ORF predictions as annotated in the *S. mansoni* genome. Figure 1 shows the alignment of SmTAL1–13. All have a C-terminal domain equivalent to the two EF-hand motifs present within SmTAL1. However the first EF-hand of SmTAL2 and 8 and the second EF-hand in SmTAL6 are missing the canonical aspartic acid residue (see Figure 1). SmTAL10 has been included in the family although it does not have a DLC domain. BLAST searches using the N-terminal EF_HAND_2 domain of SmTAL10 (residues 1–72) shows that, of all predicted *S. mansoni* gene products, it is most similar to equivalent domains in SmTALs 3, 7 and 11 (E<5e-7).

### The SmTALs display differential transcriptional profiles

**Figure 2:**
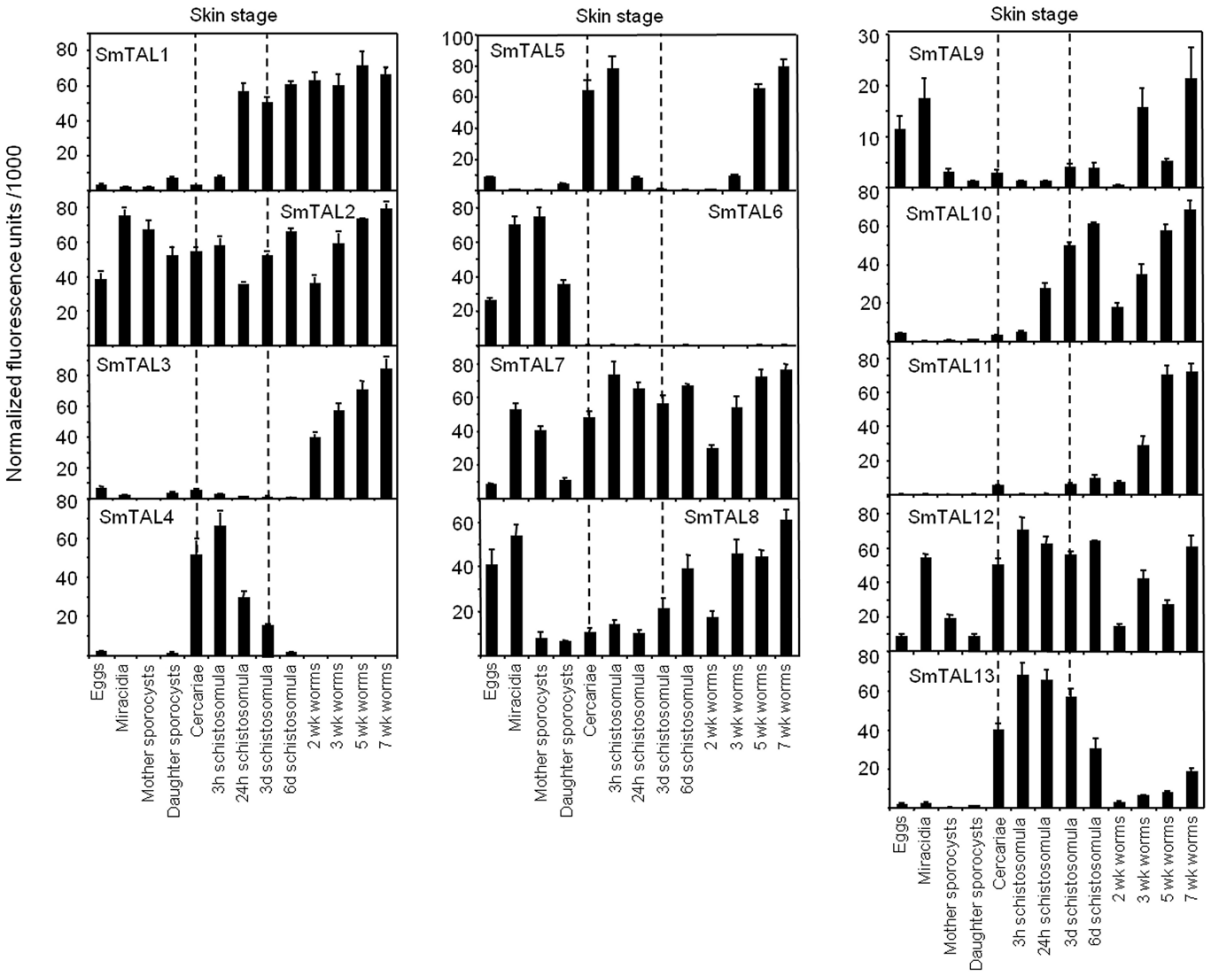
Transcription profiles of SmTALs. Profiles from the *S. mansoni* lifecycle microarray data available via Array express (31) under the experimental accession number E-MEXP-2094. Values are mean normalized fluorescence units ± sem. In primate infections, larvae remain in the skin for 2–5 days (Wilson et al. 1990). In the figure cercariae to 3 d schistosomula are denoted “skin stage”.

Using information available from DNA microarray transcriptomics database [30], [32] the mRNA abundance of each SmTAL at 13 different schistosome life-stages was deduced (Figure 2). Some members of the family are expressed throughout the life- cycle including snail stages (SmTAL2, 7, 8, 9 and 12) whilst others are predominantly expressed in the definitive host stages and especially in adult-worm (SmTAL1, 3, 10 and 11). SmTAL5 has two peaks of expression, one in cercariae and one in adult-worms. SmTAL4 and SmTAL13 are largely restricted to the cercarial and skin stages, whilst SmTAL6 expression is most marked in the miracidia and sporocysts.

### IgE and IgG4 responses to SmTALs in infected males living in an *S. mansoni* endemic area

**Figure 3:**
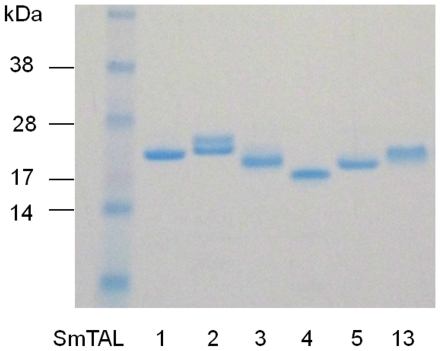
Electrophoresis of purified SmTAL proteins. 2 µg of each of the indicated proteins was run under reducing conditions on a 4–12% gradient SDS-PAGE gel and stained with Coomassie blue.

To examine the effects of different expression patterns on host antibody responses, a range of recombinant SmTAL proteins was expressed in bacteria. The antigens selected were SmTAL1 (transcribed in schistosomula and adult-worm, not egg), SmTAL3 (expressed predominantly in adult-worm, not egg), SmTAL2 (constitutively expressed throughout life-cycle including egg), SmTAL4 and 13 (transcribed predominantly in cercarial and skin stage schistosomula) and SmTAL5 (expressed in cercariae and adults). Figure 3 shows SDS-PAGE analysis of the purified recombinant proteins. SmTAL1, 3, 5 and 13 had the predicted molecular weights, SmTAL2 ran as a doublet and the N-terminus of SmTAL4 was truncated during expression to give 18 kDa rather than 20.8 (N-terminal sequencing data not shown). These recombinant antigens were used to measure SmTAL-specific antibody levels in the plasma of a cohort of *S. mansoni* infected 7–60 year old males from a high transmission area in which blood samples were donated before and 9 wks after, anti-schistosome treatment (mediated by PZQ).

**Figure 4:**
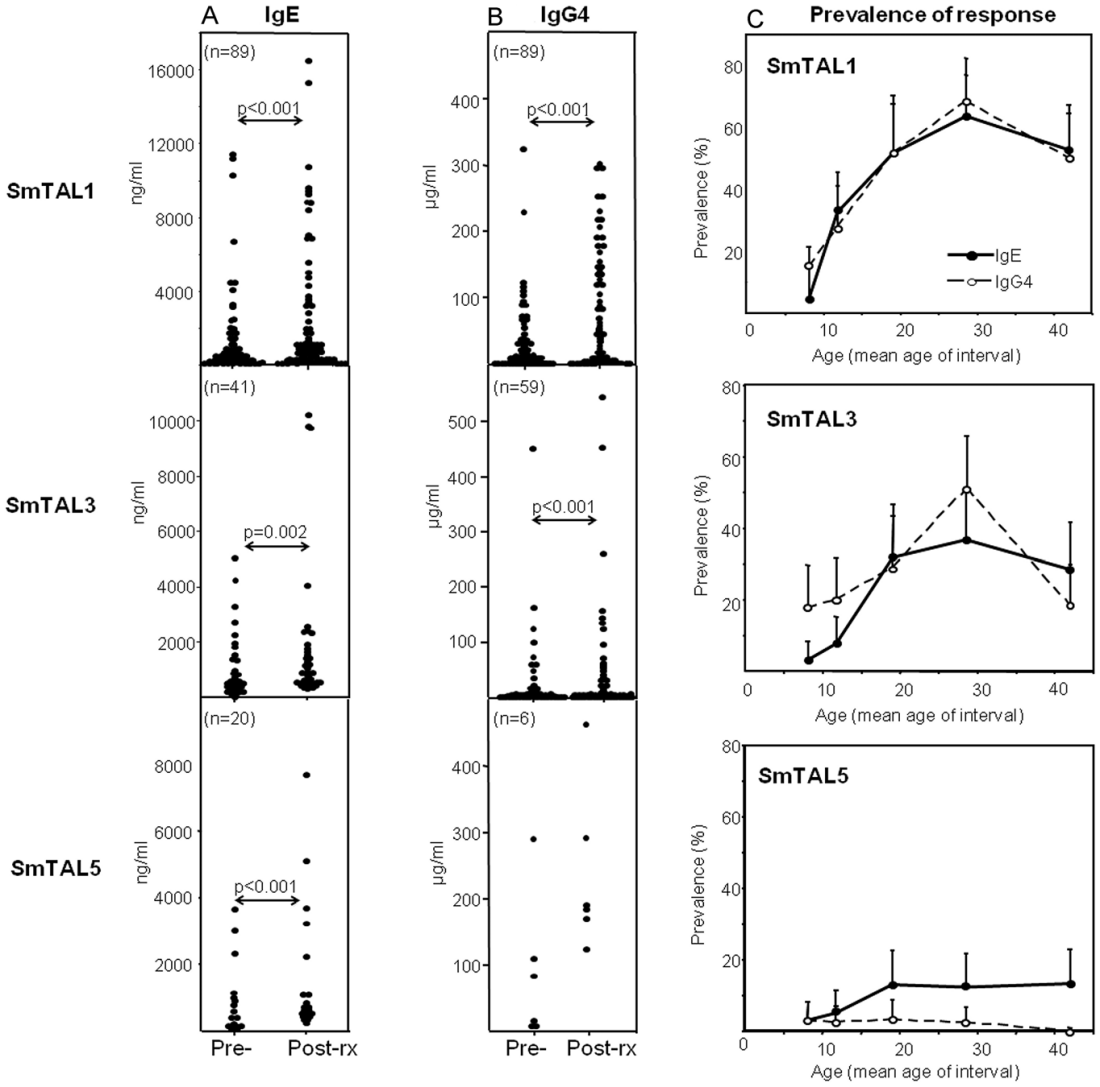
Antibody responses to SmTAL1, SmTAL3 and SmTAL5 in the *S. mansoni* infected cohort. Recombinant SmTAL1, 3 and 5 were used in ELISA to measure antigen-specific IgE (**A**) or IgG4 (**B**) before and after praziquantel treatment in 200 males infected with *S. mansoni*. Only individuals whose levels exceeded the seropositive threshold (mean+3xSD uninfected controls) for each response are graphed. For the whole cohort, the prevalence of each response (**C**) is shown in 5 age groups, 7–9 (n = 36), 10–14 (n = 43), 15–24 (n = 35), 25–34 (n = 43) and 35–60 (n = 43) Shown is % seropositive for each group after treatment +95% confidence intervals.

**Figure 5:**
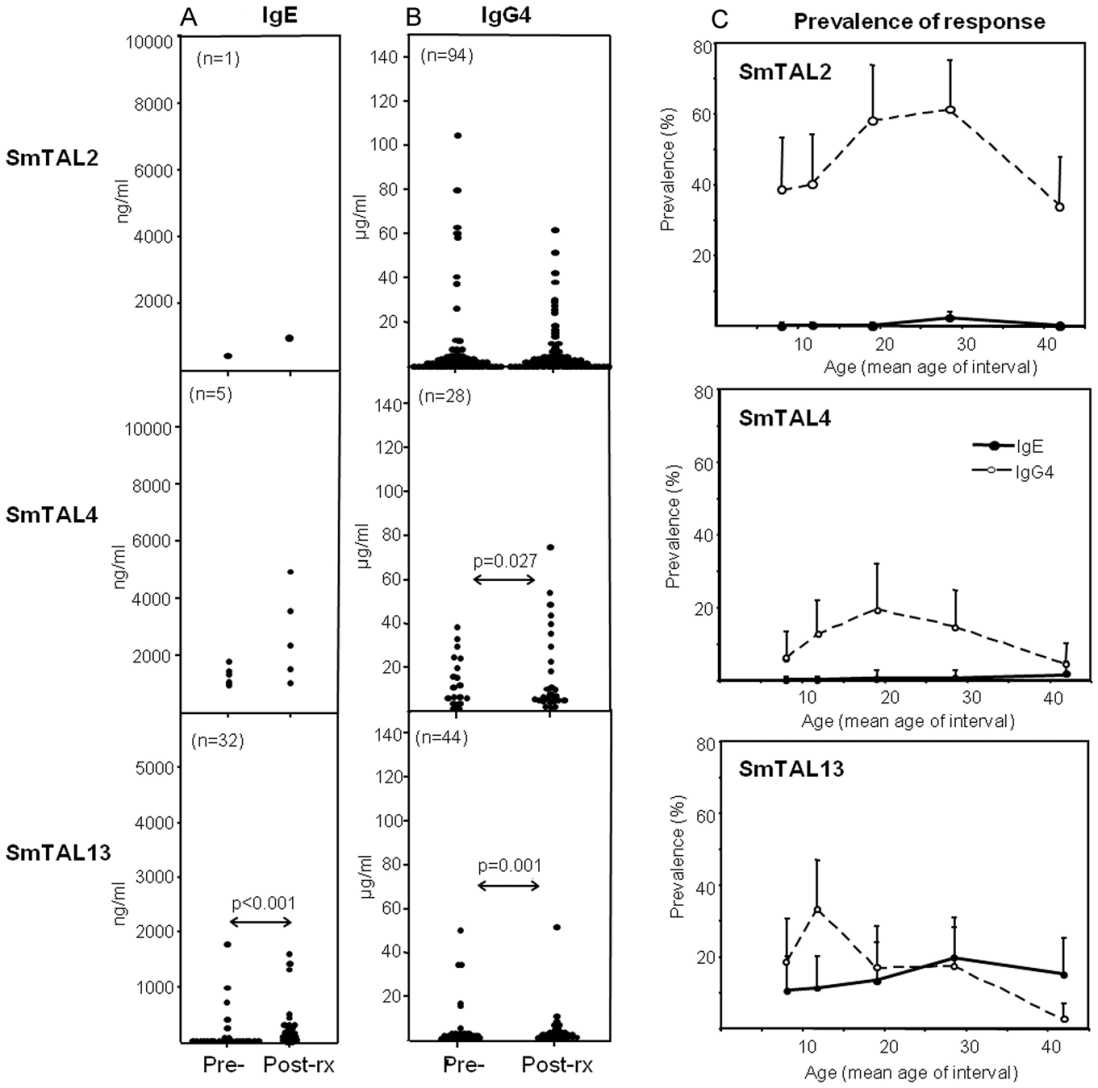
Antibody responses to SmTAL2, SmTAL4 and SmTAL13 in the *S. mansoni* infected cohort. Recombinant antigens were used in ELISA to measure IgE (**A**) or IgG4 (**B**) before and after treatment in infected cohort. Only individuals whose levels exceeded the seropositive threshold (mean+3xSD uninfected controls) for each response are graphed. The prevalence of each response (**C**) is shown (% seropositive for each group after treatment +95% confidence intervals).

Figures 4 and 5 illustrate the IgE and IgG4 responses to the 6 antigens in the infected cohort, with Figures 4C and 5C showing the percentage positive for each response with age. For some antigens (eg. SmTAL1, 3 and 5), antibody levels increased after treatment (see Figure 4). However, the proportion of responders (% positive) did not change significantly for any of the antibody responses (data not shown) and so only post-treatment data are graphed in Figures 4C and 5C.

#### SmTAL1, SmTAL3 and SmTAL5

Many of the cohort had detectable levels of IgE to SmTAL1 (SmTAL1-IgE) and these levels increased significantly after treatment (Figure 4A). The same individuals were also positive for SmTAL1-IgG4 (Figure 4A) and levels of the two isotypes were highly correlated, before treatment (rho = 0.775, p<0.001) and after (rho = 0.750, p<0.001). Detectable IgE or IgG4 responses to SmTAL1 were rare in young children (7–9 years) but rose to over 60% of those in the 25–34 year age-group (Figure 4C). A similar pattern was noted for anti-SmTAL3 responses. Those subjects positive for SmTAL3-IgE (21% of cohort) produced correlated levels of SmTAL-IgG4 (rho = 0.598, p<0.001, post-treatment). SmTAL3-IgE was absent in the youngest, rising to 37% of the 25–34 year age-group. Similarly, the SmTAL5-IgE response appeared to develop with age. 10% of the cohort were seropositive for SmTAL5-IgE, but these were all older adolescents and adults. The response was significantly more common in those over 18 years (p>0.05). A detectable IgG4 response to SmTAL5 was rare.

#### SmTAL2, SmTAL4 and SmTAL13

The TAL expressed in the parasite egg (SmTAL2) induced very different responses to those restricted to schistosomula and adult-worms (Figure 5). Only one individual was seropositive for SmTAL2-IgE, yet 47% of subjects produced detectable SmTAL2-IgG4 and this was highly prevalent in all age groups, including the 7–9 year old boys (Figure 5C).

Responses to those SmTALs restricted to cercarial and skin-stage schistosomula (SmTAL4 and 13) are also shown in Figure 5. The IgE response to SmTAL4 was virtually absent in this cohort, whilst low concentrations of IgG4 binding to SmTAL4 were detected in some (Figure 5C). Concentrations of SmTAL13-IgE were generally low, but consistently present at 10–15% of all age groups. IgG4 to SmTAL13 was detected in some younger individuals but became less common in adults.

### SmTAL4 is restricted to the cercarial tail

**Figure 6:**
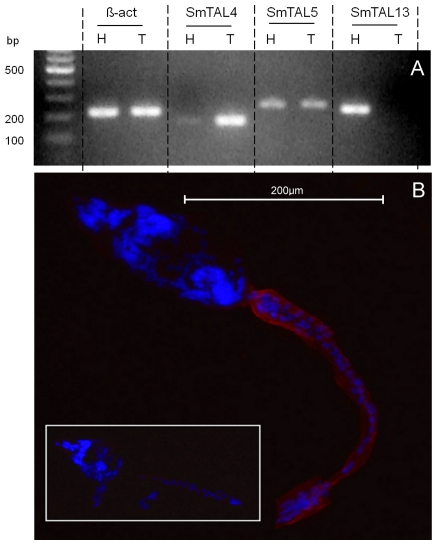
Expression of SmTAL4 in cercarial tail only. For PCR analysis (**A**), total RNA from isolated from heads (H) and tails (T) after mechanical separation and used to prepare cDNA for use with specific primers to generate amplicons for Sm ß-actin (203 bp), SmTAL4 (152 bp), SmTAL5(228 bp) and SmTAL13(206 bp). Products were separated on a 2% agarose gel and detected with ethidium bromide. For immunostaining (**B**) 8 µ sections of frozen sections of whole cercariae were fixed and stained with rat anti-SmTAL4 antiserum and TRITC -anti-rat antibody (red). Nuclei were counterstained with DAPI (blue). In the negative control (insert) anti- SmTAL4 antiserum was pre-absorbed with recombinant SmTAL4.

Expression of SmTAL4, SmTAL5 and SmTAL13 all peak in the cercarial/skin-stage phase of the schistosome life-cycle (see Figure 2). During skin penetration, the cercarial head separates from its tail to become the parasitic schistosomulum. The heads and tails are very different in composition and we considered that TAL expression might differ in the two tissues. Therefore, cercariae were mechanically separated into heads and tails and RNA isolated from each of the two samples for RT-PCR analysis. Figure 6A shows that whilst SmTAL5 is transcribed in both heads and tails, SmTAL4 is restricted to the tails and SmTAL13 to the heads. A faint SmTAL4 band in the heads lane would suggest some expression in this tissue, however SmTAL4 is so abundant in tails that contamination of head preparations with a small number of tails is the most likely explanation (data not shown). Staining of whole cercariae with anti-SmTAL4 anti-sera shows that the antigen is located in the tegument of tail only (Figure 6B), confirming the RT-PCR results.

## Discussion

TAL proteins have been identified in *Schistosoma*, *Fasciola* and *Clonorchis* species [17], [28]. Their function is not known, but from the localization studies done thus far [12], [24]–[28], it seems likely that they all have roles within the tegument. This structure is a complex vesicle-rich, syncytial layer, characteristic of parasitic platyhelminths. Bounded by a heptalaminate membrane, the tegument forms the outer covering of schistosomula and adult schistosomes and serves as their interface with the host. Hoffmann and Strand [19] showed that SmTAL3 was associated with dynein. They suggested that TAL molecules may be involved in the transport of vesicles within the tegumental cytoplasm [39], probably within dynein motor complexes. Braschi *et al.* [40] later showed that SmTAL1 and 2 were closely associated with the membranes covering the tegument of adult-worms. They speculated that the TALs were in the electron dense layer immediately below, and attached to, the underside of the membranes [40], [41], components of a cytoskeletal assembly that was probably involved in shuttling vesicles into the tegument surface [42]. SmTAL transcripts do not contain signal peptides and there is no evidence that SmTALs are secreted. However, there are proteomic studies showing that these molecules can be liberated from cercariae invading human skin [43] and adult-worms cultured *in vitro* [44]. The SmTALs may not be on the surface, but some are very close and may be exposed to the host immune system under certain circumstances. For example, this could occur when the tegument is disrupted during chemotherapy [45] or by the death of the parasite during life-cycle progression within the definitive host or during the extensive re-organisation of the surface when cercariae transform into schistosomula [46].

A large number of known molecular allergens contain the EF-hand domain (www.allergome.org/) and it is possible that many members of TAL protein family contain IgE epitopes. We have identified 13 TALs in the *S. mansoni* genome and examined their developmental expression profiles. We propose that human antibody responses to these allergen-like molecules reflect antigen exposure within the mammalian host and this is influenced by when and where they are expressed.

As noted previously [29], SmTAL1 and SmTAL3 are highly expressed in the adult stages of the parasite. It has been suggested that it is the death of worms, after years in situ, that immunizes and boosts with such antigens [47]. This would explain why the frequency of an IgE response to SmTAL1 and SmTAL3 steadily increases with age in a cohort living in an area highly endemic for *S. mansoni*. SmTAL1-IgE has been associated with the immunity to re-infection that is observed in endemic regions [12]–[14] and indeed the development of this age-dependent immunity over several decades, mirrors the rise in SmTAL1-IgE and SmTAL3-IgE responses. In contrast to this, some SmTALs have appreciable expression in parasite eggs and would be released continuously during chronic infection, as eggs die in host tissue. We have proposed [29] that the IgE response to these would quickly become tolerized, resulting in a predominantly IgG4 response. This could explain why IgG4 to SmTAL2 (highly expressed in eggs) was already prevalent form the earliest age studied (7–9 years old) whilst IgE was undetectable throughout.

Microarray data shows that SmTAL5 was expressed in cercariae and worms, but not in eggs. There was a significant rise in number of those seropositive for SmTAL5-IgE between children and adults, whereas SmTAL5-IgG4 was not detected. This suggests that, as with SmTAL1 and 3, the principle immunizing influence may be the occasional death of adult parasites releasing sequestered SmTAL5.

Expression of SmTAL4 and SmTAL13 were restricted to the cercarial and skin stages. Studies of *S. mansoni* in baboons suggest that schistosomula remain in the skin of primates for 2–5 days [48]. Exposure of cercarial antigens within skin would be accompanied by the immunosuppression associated with skin invasion [49]. Cercarial products have been shown to induce skin cells to release anti-inflammatory IL-10 and IL-1 receptor antagonist [50] and cercarial prostaglandins inhibit the migration of epidermal antigen presenting cells to lymph nodes [51]. These processes are likely to influence the development of antibody responses to antigens from cercariae and skin-stage schistosomula. In the current study virtually no one produced IgE to SmTAL4 and whilst IgE to SmTAL13 was detected in some, it was present at very low levels. For both larval antigens, the anti-inflammatory IgG4 was the predominant isotype.

The most striking feature of SmTAL4 was that it was expressed exclusively in the tegument of the cercarial tail. It is widely observed that the cercarial tails are released as cercariae penetrate the outer dermal layers, however, Whitfield *et al.* [52] reported that the majority of tails were retained when *S. mansoni* cercariae penetrated excised human skin *ex vivo*. It is possible, therefore, that SmTAL4 is released into the epidermis and although there was little IgE response to the molecule in the current study, some individuals were seropositive for SmTAL4-IgG4. This may be an immune response to tail, but the evidence for tail retention is by no means conclusive.

The SmTALs are not the only allergen-like protein family in *S. mansoni*. For example, the venom-like allergens (SmVALs) are another large group of antigens with a range of expression profiles [32] and unlike SmTALs, some members appear to be actively secreted by the parasite [32]. The current study shows that examining human immune responses to these families could provide important information about the natural biology of infection and of the IgE response. This can help us to understand the role of IgE in host defense and how molecular structure and exposure pattern dictate why some proteins evoke an allergic response and some do not.

## Supporting Information

Table S1 **Full-length coding region primers.** *S. mansoni* genome data [34] was used to design the listed forward and reverse primers for use in PCR cloning and sequencing of the coding regions of SmTAL4–13.
(DOCX)

Table S2 **Full-length coding region primers with added restriction sites.** The listed forward and reverse primers with restriction sites appended were used to produce amplicons for ligatation of the full-length coding regions of SmTAL4, 5 and 13 into protein expression vectors.
(DOCX)

Table S3 **Gene-specific primers for cercarial head and tail analysis.** The listed forward and reverse primers were used for gene-specific PCR on cDNA prepared from cercarial heads and tails.
(DOCX)
