# Supplementary material for: The Schistosoma mansoni Tegumental-Allergen-Like (TAL) Protein Family: Influence of Developmental Expression on Human IgE Responses
Source: PLoS Negl Trop Dis. 2012 Apr 3;6(4):e1593. doi: 10.1371/journal.pntd.0001593 (PMC3317908; doi:10.1371/journal.pntd.0001593)
Supplement: Table S1 — Full-length coding region primers. S. mansoni genome data [34] was used to design the listed forward and reverse primers for use in PCR cloning and sequencing of the coding regions of SmTAL4–13. (DOCX) [file pntd.0001593.s001.docx]

**Forward**  **Reverse**

| SmTAL4 | 5’-ATGGAACCATTCATTACTACATTTG-3’ | 5’-TCATGCATTTGTACGATAAATGA-3’ |
| --- | --- | --- |
| SmTAL5 | 5’-ATGGAACCATTTGTTAATATTTTTTTT-3’ | 5’- TCAATGTTTAGGTGTGCGCC-3’ |
| SmTAL6 | 5’-ATGGACAATTTTATTGACATATTTTCA-3’ | 5’-TTATTCATTTCTACAATCTGGTGTTT-3’ |
| SmTAL7 | 5’-ATGATAAATATGGATGCATTTGTAG-3’ | 5’-TTAAGTATTCGGTGTTTTAAACAATAG-3’ |
| SmTAL8 | 5’-ATGTTGGAAGAATTTATCAAAGCAT-3’ | 5’-TTAACCTTTTGGTGTGCGCC-3’ |
| SmTAL9 | 5’-ATGGACTCGTTTTTGGACGC-3’ | 5’-TTAAGCAGAACATGGTGTTTTCCAT-3’ |
| SmTAL10 | 5’-ATGACTGAACAAGAAATTTTACATGC-3’ | 5’-CTAACAGCAACAACATCCTGC-3’ |
| SmTAL11 | 5’-ATGGATCCATTTTTACATGCAT-3’ | 5’-TTAACACAATTGATATTGACCAGGT-3’ |
| SmTAL12 | 5’-ATGGAAAAATTAATCGAATTATTC-3’ | 5’-TCAAGAGTTATATCTTTGTCCAG-3’ |
| SmTAL13 | 5’-ATGCAAACAATTCATAAACTAGATG-3’ | 5’-CTATTCTAAATCCGGAjjATCTTGTA-3’ |
