# Supplementary material for: The Schistosoma mansoni Tegumental-Allergen-Like (TAL) Protein Family: Influence of Developmental Expression on Human IgE Responses
Source: PLoS Negl Trop Dis. 2012 Apr 3;6(4):e1593. doi: 10.1371/journal.pntd.0001593 (PMC3317908; doi:10.1371/journal.pntd.0001593)
Supplement: Table S2 — Full-length coding region primers with added restriction sites. The listed forward and reverse primers with restriction sites appended were used to produce amplicons for ligatation of the full-length coding regions of SmTAL4, 5 and 13 into protein expression vectors. (DOCX) [file pntd.0001593.s002.docx]

**Forward Reverse**

| SmTAL4 | 5’-AGGATCCATGGAACCATTCATTACT-3’ | 5’-AGAATTCTCATGCATTTGTACGATA-3’ |
| --- | --- | --- |
| SmTAL5 | 5’-AGGATCCATGGAACCATTTGTTAAT-3’ | 5’-AGAATTCTCAATGTTTAGGTGTGCG-3’ |
| SmTAL13 | 5’-AGGATCCATGCAAACAATTCATAAA-3’ | 5’-ACTCGAGCTATTCTAAATCCGGAAT-3’ |
