# Supplementary material for: The Schistosoma mansoni Tegumental-Allergen-Like (TAL) Protein Family: Influence of Developmental Expression on Human IgE Responses
Source: PLoS Negl Trop Dis. 2012 Apr 3;6(4):e1593. doi: 10.1371/journal.pntd.0001593 (PMC3317908; doi:10.1371/journal.pntd.0001593)
Supplement: Table S3 — Gene-specific primers for cercarial head and tail analysis. The listed forward and reverse primers were used for gene-specific PCR on cDNA prepared from cercarial heads and tails. (DOCX) [file pntd.0001593.s003.docx]

**Forward Reverse**

| Sm ß-actin | 5’-GACAACGAGGCCATTTATGA-3’ | 5’-TAGGTAGCCAACGGGAAGTG-3’ |
| --- | --- | --- |
| SmTAL4 | 5’-ATGATACCGAAATGGCAAGC-3’ | 5’-GGTCGTAGACCCGTTGTAGG-3’ |
| SmTAL5 | 5’-GTGATGTCCTCGGTGTCAAA-3’ | 5’-GCCATGAAGGGTCAAATGTC-3’ |
| SmTAL13 | 5’-TTCAAGGAAATGAAATGCAAGA-3’ | 5’-GCGTGACCAGTATCGACCAT-3’ |
